# Supplementary material for: Identification of Key Elements in Prostate Cancer for Ontology Building via a Multidisciplinary Consensus Agreement
Source: Cancers (Basel). 2023 Jun 8;15(12):3121. doi: 10.3390/cancers15123121 (PMC10295832; doi:10.3390/cancers15123121)
Supplement: Supplementary file 1 [file cancers-15-03121-s001.zip › cancers-2350276-supplementary.pdf]

Article

Identification of Key Elements in Prostate Cancer for Ontology Building Via a Multidisciplinary Consensus Agreement

Amy Moreno [1,*], Abhishek A. Solanki [2], Tianlin Xu [3], Ruitao Lin [3], Jatinder Palta [4], Emily Daugherty [5], Neeraj Agarwal [6], Ana Aparicio [7], Jeffrey Cadeddu [8], Ronald Chen [9], Seungtaek Choi [1], Matthew Cooperberg [10], Alan Dal Pra [11], Indra Das [12], Neil Desai [13], Dayssy Diaz Pardo [14], Weiliang Du [15], William Hall [16], Celestia Higano [17], Karen Hoffman [1], Maha Hussain [18], Amar Kishan [19], Bridget Koontz [20], Rajat Kudchadker [15], Jeff Michalski [21], Alicia Morgans [18], Himanshu Nagar [22], Louis Potter [23], Tyler Robin [24], Mihaela Rosu-Bubulac [25], Howard Sandler [26], Neal Shore [27], Cora Sternberg [28], Rahul Tendulkar [29], Ying Xiao [30], James Yu [31], Zachary Zumsteg [26], David Hong [32], Julian Hong [33], Sophia C. Kamran [34], Evangelia Katsoulakis [35], Kristy Brock [15], Mary Feng [33], Clifton Fuller [1], Charles Mayo [36]

1. Department of Radiation Oncology, University of Texas MD Anderson Cancer Center, Houston, TX 77030-4009, USA; cdfuller@mdanderson.org (C.F.)
2. Department of Radiation Oncology, Loyola Medicine—MacNeal Hospital, Berwyn, IL 60402, USA; asolanki@luc.edu
3. Department of Biostatistics, University of Texas MD Anderson Cancer Center, Houston, TX 77030-4009, USA; txu2@mdanderson.org (T.X.); rlin@mdanderson.org (R.L.)
4. Department of Medical Physics, Virginia Commonwealth University, Richmond, VA 23284, USA; jatinder.palta@vcuhealth.org
5. Department of Radiation Oncology, University of Cincinnati College of Medicine, Cincinnati, OH 45267, USA; daugheec@ucmail.uc.edu
6. Department of Oncology, University of Utah Huntsman Cancer Institute, Salt Lake City, UT 84112, USA
7. Department of Genitourinary Medical Oncology, University of Texas MD Anderson Cancer Center, Houston, TX 77030-4009, USA
8. Department of Urology, University of Texas Southwestern Medical Center, Dallas, TX 75390, USA
9. Department of Radiation Oncology, University of Kansas Medical Center, Kansas City, Kansas 66160, USA
10. Department of Urology, University of California San Francisco, San Francisco, CA 93701, USA
11. Department of Radiation Oncology, University of Miami Miller School of Medicine, Miami, FL 33136, USA
12. Department of Radiation Oncology, Northwestern University Feinberg School of Medicine, Chicago, IL 60611, USA
13. Department of Radiation Oncology, University of Texas Southwestern Medical Center, Dallas, TX 75390, USA
14. Department of Radiation Oncology, Ohio State University Comprehensive Care Center, Columbus, OH 43210, USA
15. Department of Imaging Physics, University of Texas MD Anderson Cancer Center, Houston, TX 77030-4009, USA; kkbrock@mdanderson.org (K.B.)
16. Department of Radiation Oncology, Medical College of Wisconsin, Milwaukee, WI 53226, USA
17. Department of Medicine and Urology, University of Washington, Fred Hutchinson Cancer Research Center, Seattle, WA 98195, USA
18. Department of Hematology and Oncology, Northwestern University Feinberg School of Medicine, Chicago, IL 60611, USA

[19] Department of Radiation Oncology, University of California, Los Angeles, CA 90095, USA
[20] Department of Radiation Oncology, East Carolina University, Greenville, NC 27834, USA
[21] Department of Radiation Oncology, Washington University School of Medicine, St. Louis, MO 63110, USA
[22] Department of Radiation Oncology, Weill Cornell Medicine, New York City, NY 10065, USA
[23] Department of Radiation Oncology, Northwell Health, New York, NY 10075, USA
[24] Department of Radiation Oncology, University of Colorado Denver School of Medicine, Aurora, CO 80045, USA
[25] Department of Radiation Oncology, Virginia Commonwealth University, Richmond, VA 23298-0058
[26] Department of Radiation Oncology, Cedars-Sinai Medical Center, Los Angeles, CA 90048, USA
[27] Department of Urology, Atlantic Urology Clinics, Myrtle Beach, SC 29572, USA
[28] Department of Medical Oncology, Weill Cornell Medicine, New York City, NY 10065, USA
[29] Department of Radiation Oncology, Cleveland Clinic Cancer Center, Cleveland, OH 44106, USA
[30] Department of Radiation Oncology, University of Pennsylvania, Perelman School of Medicine, Philadelphia, PA 19104, USA
[31] Department of Radiation Oncology, Trinity Health of New England, Hartford, CT 06105, USA
[32] Department of Radiation Oncology, University of Southern California, Los Angeles, CA 90089, USA; david.hong@med.usc.edu
[33] Department of Radiation Oncology, University of California San Francisco, San Francisco, CA 93701, USA; julian.hong@ucsf.edu (J.H.); mary.feng@ucsf.edu (M.F.)
[34] Department of Radiation Oncology, Massachusetts General Hospital, Boston, MA 02129, USA; sophia_kamran@post.harvard.edu
[35] Department of Radiation Oncology, James A Haley VA Medical Center, Tampa, FL 33612, USA; ekatsoulakis@usf.edu
[36] Department of Radiation Physics, University of Michigan, Ann Arbor, MI 48109, USA; cmayo@med.umich.edu
* Correspondence: akmoreno@mdanderson.org; Tel.: +1-281-728-6162
